# Association Between Long-Term Testosterone Exposure and Major Adverse Cardiovascular Events in Aging Men

**DOI:** 10.1210/jendso/bvaf156

**Published:** 2025-10-07

**Authors:** Paul J Connelly, Samuel Owusu Achiaw, Jocelyn M Friday, Frederick K Ho, Claudia Geue, Sandosh Padmanabhan, Jill P Pell, Daniel F Mackay, Ruth Dundas, Tran Q B Tran, Denise Brown, Claire E Hastie, Michael Fleming, Alan Stevenson, Clea du Toit, Jim Lewsey, Christian Delles

**Affiliations:** Department of Endocrinology & Diabetes, Queen Elizabeth University Hospital, Glasgow G51 4TF, UK; School of Health and Wellbeing, University of Glasgow, Glasgow G12 8TB, UK; School of Health and Wellbeing, University of Glasgow, Glasgow G12 8TB, UK; School of Health and Wellbeing, University of Glasgow, Glasgow G12 8TB, UK; School of Health and Wellbeing, University of Glasgow, Glasgow G12 8TB, UK; School of Cardiovascular & Metabolic Health, University of Glasgow, Glasgow G12 8TB, UK; School of Health and Wellbeing, University of Glasgow, Glasgow G12 8TB, UK; School of Health and Wellbeing, University of Glasgow, Glasgow G12 8TB, UK; School of Health and Wellbeing, University of Glasgow, Glasgow G12 8TB, UK; School of Cardiovascular & Metabolic Health, University of Glasgow, Glasgow G12 8TB, UK; School of Health and Wellbeing, University of Glasgow, Glasgow G12 8TB, UK; School of Health and Wellbeing, University of Glasgow, Glasgow G12 8TB, UK; School of Health and Wellbeing, University of Glasgow, Glasgow G12 8TB, UK; School of Health and Wellbeing, University of Glasgow, Glasgow G12 8TB, UK; The Living Laboratory, University of Glasgow, Glasgow G12 8TB, UK; School of Health and Wellbeing, University of Glasgow, Glasgow G12 8TB, UK; School of Cardiovascular & Metabolic Health, University of Glasgow, Glasgow G12 8TB, UK

**Keywords:** testosterone exposure, hypogonadism, major adverse cardiovascular events, retrospective cohort study

## Abstract

**Context:**

Hypogonadism is a common endocrine disorder in aging men, associated with adverse cardiometabolic outcomes. Concerns about the cardiovascular (CV) safety of testosterone, an important therapy option for the condition, may be disproportionately influencing treatment decisions.

**Objective:**

This work aimed to investigate the association between long-term testosterone therapy and major adverse CV events (MACE) in men aged 51 years and older.

**Methods:**

This retrospective cohort study used linked health data from the National Health Service Greater Glasgow and Clyde population, accessed via the West of Scotland Safe Haven. Men aged 51 years and older as of January 1, 2012, were included. Testosterone exposure was defined as having at least a 2-year interval between the first and last prescription during a 5-year exposure window (2012-2016). Individuals were followed from January 1, 2017, to December 31, 2022. The primary outcome was time to first MACE, defined as a composite of acute myocardial infarction, unstable angina, stroke, heart failure, or CV death. Cox proportional hazards models were used to estimate associations, adjusting for age, ethnicity, socioeconomic deprivation, and comorbidities.

**Results:**

The study included 440 testosterone-exposed and 136 051 unexposed men. Testosterone exposure was associated with a 54% increased risk of MACE in the unadjusted analysis (hazard ratio [HR]: 1.54; 95% CI, 1.18-2.00), and a 55% increased risk after adjustment (HR: 1.55; 95% CI, 1.19-2.01).

**Conclusion:**

In this real-world cohort, long-term testosterone therapy was associated with increased CV risk. While recent trials inform short- to medium-term CV safety, this study underscores the need for more longer-term data to fully ascertain the effect of testosterone therapy.

Hypogonadism is a common endocrine disorder in aging men, characterized by decreased testosterone production and impaired spermatogenesis [1]. The crude incidence rate of this condition in men older than 40 years is 12.3 per 1000 person-years, and this increases significantly with age [2]. Hypogonadism adversely affects sexual, metabolic, and musculoskeletal health and is associated with increased mortality [3, 4]. Testosterone therapy has demonstrated efficacy in improving sexual function, anemia, physical fitness, bone mineral density, and depressive symptoms [5-7].

Despite these benefits, concerns about the cardiovascular (CV) safety of testosterone therapy may be unduly influencing treatment decisions in older hypogonadal men [3, 8, 9]. These concerns stemmed from observational studies linking testosterone use to cardiovascular disease (CVD) and the early termination of a placebo-controlled trial due to increased CV events in older men with comorbidities [10-12]. The resulting US Food and Drug Administration warnings have had a lasting effect, leading to heightened caution in prescribing testosterone therapy [13].

Recent studies have provided reassurance regarding the cardiovascular safety of testosterone therapy. Both the Testosterone Replacement Therapy for Assessment of Long-term Vascular Events and Efficacy Response in Hypogonadal Men (TRAVERSE) randomized trial [14] and the Testosterone and Cardiovascular Events Study (TestES) patient-level meta-analysis [9] reported no significant increase in major adverse cardiovascular events (MACE) with testosterone treatment in the short to medium term [9, 14]. However, questions remain about the long-term CV implications of testosterone therapy, particularly in real-world settings.

Consequently, this study aims to investigate the association between testosterone therapy and CV outcomes in a large, regional cohort, with an extended follow-up period and varied testosterone formulations used in routine clinical practice.

## Materials and Methods

### Study Design and Population

This retrospective cohort study used linked datasets from the Safe Haven database of the National Health Service (NHS) Greater Glasgow and Clyde (GGC) Health Board to estimate the association between testosterone therapy and CV outcomes. The cohort comprised adult men aged 51 years and older, residing within the NHS GGC area as of January 1, 2012. The exposed group comprised men who received testosterone therapy (likely for the treatment of primary or secondary hypogonadism) for a minimum of 2 years over a 5-year window prior to follow-up (ie, from January 1, 2012 to December 31, 2016). This 2-year threshold was chosen to reflect sustained testosterone use, distinguishing long-term therapy from intermittent or trial use. This was defined by the interval between the first and last testosterone prescription (regardless of formulation, dosage, or mode of administration) during the 5-year window. Testosterone prescriptions were identified using British National Formulary codes through the OpenPrescribing data portal [15, 16]. Testosterone formulations were classified by the first recorded prescription in the dataset (ie, from January 1, 2012)

The control cohort (unexposed group) consisted of men in the same age group and region who did not receive any testosterone prescriptions during this time period. Both groups were followed up from January 1, 2017 until the first MACE, non-CVD death, or the end of the study follow-up period December 31, 2022, whichever occurred first. Non-CVD deaths were treated as censoring events in the Cox models. Individuals (in both groups) with a documented history of heart failure, unstable angina, myocardial infarction, or stroke before the start of follow-up were excluded.

### Data Extraction

The data for the cohort were identified, linked, pseudonymized, and extracted from the West of Scotland Haven using the Community Health Index numbers. The Community Health Index is a unique patient identifier in Scotland that enables the linkage of health-care contacts across various settings [17], allowing for the accurate tracking of exposures and outcomes over time. The West of Scotland Safe Haven provided individual-level pseudonymized patient health data for individuals served by NHS GGC. The dataset included demographic information, hematology and biochemistry laboratory results, community-dispensed medications, hospital admissions, procedural codes, and mortality records (Supplementary Table S1 [18]). Key variables, such as age, sex, ethnicity, socioeconomic status, multimorbidity, number and duration of testosterone prescriptions, CV risk factors, and prior CVD, were defined. It should be noted that ethnicity data were obtained from hospitalization records. Individuals who were never admitted were classified as “missing” ethnicity data. Coding for chronic conditions relating to comorbidities is detailed in Supplementary Table S2 [18]. Data extraction and linkage were performed by the West of Scotland Safe Haven Service. All data were stored and handled within the secure NHS Safe Haven platform.

### Ethical Approval

Delegated research ethics approval was granted for linkage to NHS patient data by the Local Privacy and Advisory Committee at NHS Greater Glasgow and Clyde. Cohorts and deidentified linked data were prepared by the West of Scotland Safe Haven at NHS GGC, a trusted research environment (IRAS Project ID 321198, REC reference 22/WS/1063).

### Outcomes

The primary outcome was time to the first MACE, defined by a composite of 5 events: acute myocardial infarction, unstable angina, heart failure, stroke, and CV death [19]. International Statistical Classification of Diseases and Related Health Problems 10th Revision (ICD-10) codes for these conditions are provided in Supplementary Table S3 [18].

CVD deaths were identified using ICD-10 codes I00-I99. The date and underlying cause of death were obtained from National Records Scotland. Baseline characteristics, recorded as of January 1, 2012, included age, sex, ethnicity, and quintiles of the Scottish Index of Multiple Deprivation (SIMD) 2016. Comorbidities preceding any first MACE event or non-CVD death were identified based on recorded diagnoses from the health-care records. Comorbidities were dichotomized as either being present or not. Diagnostic lists were adapted from the CALIBER phenotypes [20]. Patients without a recorded diagnosis were presumed to be free of that condition.

### Statistical Analysis and Reporting

Descriptive statistics summarized patient demographics, comorbidities, and cardiovascular outcomes. Continuous variables were expressed as means with SDs, while categorical variables were presented as frequencies and percentages. Differences in proportions between the exposed and unexposed groups for the first MACE and non-CV deaths were assessed using *t* tests for proportions. The association between testosterone exposure and time to the first MACE was assessed using Cox proportional hazards regression models. This was a complete case analysis, with the adjusted regression models including only individuals with observations for all covariates. Covariates included in the models were age, CV risk factors, socioeconomic deprivation (as measured by SIMD), ethnicity, and comorbidities such as peripheral vascular disease, hypertension, dementia, pulmonary disease (chronic obstructive pulmonary disease, asthma, bronchiectasis), rheumatic and connective tissue diseases, liver disease, severe renal disease, diabetes, hyperthyroidism, hypothyroidism, and malignancy. The covariates included in the analysis were selected based on whether they were potential confounders or potentially related to the outcome of interest. Hazard ratios (HRs) were presented with 95% CIs, and *P* values below .05 were considered statistically significant. We also conducted sensitivity and subgroup analyses. A sensitivity analysis was performed to assess the effect of ethnicity on study results (given the aforementioned challenges with the ethnicity data). First, ethnicity was omitted from the list of covariates, and then another model was analyzed that included ethnicity as a covariate, but included only the categories “White” and “non-White.” In a subgroup analysis, the association between different formulations of testosterone (transdermal and injectable) and MACE was assessed. All analyses were conducted using R (version 4.0; R Foundation for Statistical Computing) and Stata (version 18; StataCorp LLC). This study was reported following the Strengthening the Reporting of Observational Studies in Epidemiology (STROBE) guidelines [21].

## Results

### Baseline Characteristics

The study cohort included 440 testosterone-exposed men and 136 051 unexposed men, all aged 51 years and older as of January 1, 2012. The mean age of the testosterone-exposed group was 61.5 years (SD 7.1), compared to 62.8 years (SD 8.7) in the unexposed group. The majority of participants in both groups were White (84.8% in the testosterone-exposed group and 75.1% in the unexposed group). Non-White ethnicities were reported in 4.3% and 2.7%, respectively, while unknown or “refused to state” (5.7% in exposed vs 10.4% in unexposed) and missing ethnicity (5.2% in exposed vs 11.8% in unexposed) were found to be more common in participants in the unexposed group. Socioeconomic deprivation, as measured by SIMD 2016 quintiles, was similar across both groups. In the testosterone-exposed group, 34.1% of participants were from the most deprived quintile compared to 32.9% in the unexposed group. The least deprived quintile comprised 19.6% and 21.8% of the exposed and unexposed groups, respectively.

In the testosterone-exposed group, the most common formulation of the first testosterone prescription (during the 5-year exposure window, ie, 2012-2016) was injectable/intramuscular testosterone (n = 247, 56.1%). Transdermal formulations, including gel and patches, were prescribed to 179 men (40.7%). A smaller proportion of individuals received oral testosterone (n = 14, 3.2%). Baseline characteristics are shown in Table 1.

**Table 1.** Baseline characteristics

Baseline characteristic	Testosterone-exposed (n = 440)	Unexposed (n = 136 051)
Mean age, y	61.5 (SD 7.1)	62.8 (SD 8.7)
Ethnicity, n (%)		
White	373 (84.8%)	102 143 (75.1%)
Other	19 (4.3%)	3720 (2.7%)
Unknown	25 (5.7%)	14 126 (10.4%)
Missing	23 (5.2%)	16 062 (11.8%)
Socioeconomic deprivation (SIMD) quintiles, n (%)		
1 (most deprived)	150 (34.1%)	44 733 (32.9%)
2	71 (16.1%)	21 970 (16.2%)
3	63 (14.3%)	18 320 (13.5%)
4	63 (14.3%)	19 949 (14.7%)
5 (least deprived)	86 (19.6%)	29 659 (21.8%)
Missing	7 (1.6%)	1420 (1.0%)
Comorbidities, n (%)		
Respiratory diseases (COPD, asthma, and bronchitis)	62 (14.1%)	13 912 (10.2%)
Dementia	16 (3.6%)	5744 (4.2%)
Diabetes with end-organ failure	19 (4.3%)	1643 (1.2%)
Diabetes—uncomplicated	96 (21.8%)	14 282 (10.5%)
Hypertension	106 (24.1%)	22 043 (16.2%)
Thyroid diseases (hyperthyroidism and hypothyroidism)	14 (3.2%)	1267 (0.93%)
Malignancy	97 (22.1%)	25 197 (18.5%)
Metastatic disease	18 (4.1%)	7212 (5.3%)
Mild liver disease	25 (5.7%)	5000 (3.7%)
Peripheral vascular disease	32 (7.3%)	7847 (5.8%)
Renal disease	50 (11.4%)	7478 (5.5%)
Rheumatic/Connective tissue disease	15 (3.4%)	1763 (1.3%)
Severe liver disease	11 (2.5%)	2109 (1.6%)

Abbreviations: COPD, chronic obstructive pulmonary disease; SIMD, Scottish Index of Multiple Deprivation.

### Comorbidities Before First Major Adverse Cardiovascular Events

Comorbidities were prevalent in both groups, but the testosterone-exposed group had a higher proportion of certain conditions. Hypertension was present in 24.1% of the testosterone-exposed group compared to 16.2% of the unexposed group. Similarly, diabetes without complications was reported in 21.8% of the testosterone-exposed group vs 10.5% of the unexposed group. Other differences included renal disease (11.4% vs 5.5%) and respiratory conditions (14.1% vs 10.2%). Last, malignancy was more frequently observed in the testosterone-exposed group (22.1%) compared to the unexposed group (18.5%). For the exposed group, the mean testosterone exposure time from first recorded prescription during the exposure period to the end of study follow-up was 8.3 years (SD: 2.8 years). The mean testosterone exposure time during the exposure window, that is, before the start of follow-up, was 4.15 years (SD: 0.82 years) (Table 2).

**Table 2.** Duration of testosterone exposure before follow-up and throughout the study period (in years) in the testosterone-exposed group

	Mean (SD)	Median	5th Percentile	95th Percentile
Testosterone exposure before follow-up	4.15 (0.82)	4.51	2.31	4.91
Testosterone exposure through follow-up period (from start of study to end of follow-up)	8.28 (2.78)	9.46	2.86	10.88

### Cardiovascular Events

During the follow-up period, 56 (12.7%; 95% CI, 9.6%-15.8%) testosterone-exposed men experienced a first MACE event, compared to 11 662 (8.6%; 95% CI, 8.4%-8.7%) of the unexposed men (*P* = 0.002). In terms of non-CV deaths, 75 (17.0%; 95% CI, 13.5%-20.1%) deaths occurred in the testosterone-exposed group, compared to 19 919 (14.6%; 95% CI, 14.5%-14.8%) in the unexposed group (*P* = .154).

**Figure 1.**
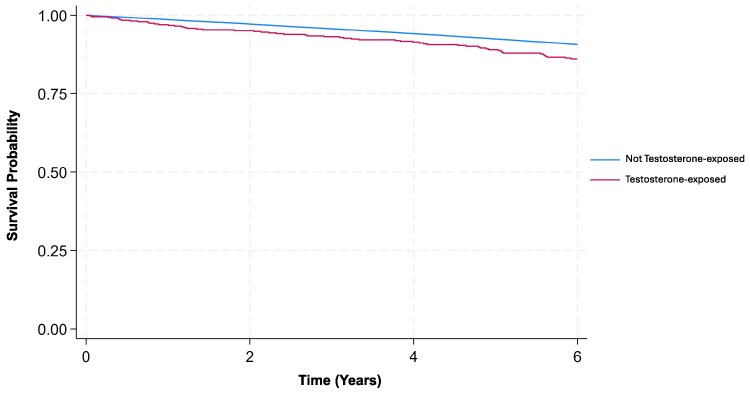
Kaplan-Meier estimates comparing time to first major adverse cardiovascular events between “Testosterone-exposed” (red) and “Not testosterone-exposed” over the follow-up period (years).

The association between testosterone exposure and the risk of MACE was evaluated using Cox proportional hazards models (Table 3, Fig. 1). In the unadjusted model, testosterone exposure was associated with a 54% increased risk of MACE (HR: 1.54; 95% CI, 1.18-2.00; *P* = .001). Following adjustment for age, socioeconomic status, ethnicity, and comorbidities, testosterone exposure was associated with a 55% increased risk of MACE (HR: 1.55; 95% CI, 1.19-2.01; *P* = .001).

**Table 3.** Cox proportional hazards model of testosterone exposure and major adverse cardiovascular events

Unadjusted model							
Covariates	Hazard ratio	95% CI		*P*	No. of events	Total person-time at risk, in person-y	Average time at risk, in y
Unexposed group (n = 136 051)	Ref.				11 662	720 107.58	5.29
Testosterone-exposed (n = 440)	1.540	1.184	2.001	.001	56	2252.70	5.12

Adjusted model							
Covariates	Hazard ratio	95% CI		*P*	No. of events	Person-time at risk, in person-y	Average time at risk, in y
Unexposed group (n = 118 757)	Ref.				11 303	619 254.81	5.21
Testosterone-exposed (n = 410)	1.547	1.189	2.012	.001	56	2087.56	5.09

Hazard ratios generated for the other covariates in the adjusted models have been omitted to avoid a Table 2 fallacy interpretation. Covariates included in the adjusted models were: age, SIMD quintiles (1 [most deprived], 2, 3, 4, 5 [least deprived], comorbidities (COPD/asthma/bronchiectasis, dementia, diabetes-uncomplicated, diabetes with end-organ failure, hypertension, hyperthyroidism, hypothyroidism, malignancy, metastatic disease, mild liver disease, peripheral vascular disease, renal disease, rheumatic/connective tissue disease, severe liver disease), ethnicity (White, non-White, unknown).

Abbreviations: COPD, chronic obstructive pulmonary disease; Ref., reference; SIMD, Scottish Index of Multiple Deprivation.

### Sensitivity and Subgroup Analysis

Multiple sensitivity analyses were performed to assess the effect of ethnicity on the study results. For the first model that omitted ethnicity from the list of covariates, a 63% increased risk in MACE for the testosterone-exposed group compared to the unexposed group (HR: 1.63; 95% CI, 1.25-2.17; *P* = .000) was observed. Including ethnicity but including only the categories “White” and “non-White” resulted in a 51% increased risk of MACE in the testosterone-exposed group compared to the unexposed group (HR: 1.51; 95% CI, 1.15-1.978; *P* = .003).

In the subgroup analysis, both in unadjusted and adjusted models, only transdermal testosterone was significantly associated with increased MACE risk; the association with injectable testosterone was not statistically significant. The results of the subgroup analysis are shown in Table 4.

**Table 4.** Subgroup analysis: Cox model of testosterone exposure and major adverse cardiovascular events by testosterone formulation (injectable vs transdermal)

Unadjusted model for injectable testosterone							
Covariates	Hazard ratio	95% CI		*P*	No. of events	Total person-time at risk, in person-y	Mean time at risk, in y
Unexposed group (n = 136 051)	Ref.				11 662	720 107.58	5.29
Testosterone-exposed (n = 247)	1.432	0.995	2.061	.053	29	1254.78	5.08

Adjusted model for injectable testosterone							
Covariates	Hazard ratio	95% CI		*P*	No. of events	Total person-time at risk, in person-y	Mean time at risk, in y
Unexposed group (n = 118 757)	Ref.				11 303	619 254.80	5.21
Testosterone-exposed (n = 229)	1.429	0.992	2.058	.055	29	1162.59	5.08

Unadjusted model for transdermal testosterone							
Covariates	Hazard ratio	95% CI		*P*	No. of events	Total person-time at risk, in person-y	Mean time at risk, in y
Unexposed group (n = 136 051)	Ref.				11 662	720 107.58	5.29
Testosterone-exposed (n = 179)	1.668	1.127	2.470	.011	25	926.33	5.18

Adjusted model for transdermal testosterone							
Covariates	Hazard ratio	95% CI		*P*	No. of events	Total person-time at risk, in person-y	Mean time at risk, in y
Unexposed group (n = 118 757)	Ref.				11 303	619 254.80	5.21
Testosterone-exposed (n = 168)	1.672	1.129	2.476	.010	25	860.37	5.12

Abbreviation: Ref., reference.

## Discussion

The findings of this retrospective cohort study suggest that testosterone therapy is associated with an increased risk of MACE. In the adjusted model, testosterone exposure was linked with a 55% higher risk of MACE compared to the unexposed cohort (HR: 1.55; 95% CI, 1.19-2.01). Similar findings were observed in the sensitivity analyses.

The findings from this study are consistent with several retrospective analyses that reported increased CV risk following testosterone therapy. Finkle et al [10] reported a significant increase in the risk of acute nonfatal myocardial infarction in older men within 90 days of initiating testosterone therapy. Similarly, a study of 8709 hypogonadal men undergoing coronary angiography reported a 29% increased risk of all-cause mortality, myocardial infarction, and ischemic stroke among testosterone-treated individuals [11]. Although non-CV deaths were more frequent in the testosterone-exposed group in our analysis, the difference did not reach statistical significance. More recently, an analysis of UK general practice data observed an elevated risk of CV events during the first 2 years of testosterone use [22].

In contrast, other large retrospective studies have described neutral or protective associations between testosterone therapy and CV outcomes. A retrospective analysis of older Medicare beneficiaries found no association between intramuscular testosterone and myocardial infarction over a mean follow-up period of 2.5 years [23]. Likewise, in a retrospective study of 8808 hypogonadal men, testosterone therapy was associated with lower CV risk over a median follow-up of 3.4 years (HR 0.67; 95% CI, 0.62-0.73) [24]. Several additional studies have reported reduced rates of myocardial infarction, stroke, or all-cause mortality in men treated with testosterone, particularly when testosterone levels were normalized or therapy was sustained over time [25-31].

Heterogeneity in study design likely contributes to the divergent findings across these analyses. Key differences include whether testosterone levels were monitored and normalized, the duration and consistency of exposure, and the inclusion of high-risk subgroups such as men with type 2 diabetes or prostate cancer. Follow-up periods varied, with many of these studies having shorter follow-up periods than the present study, as did outcome definitions and the scope of covariate adjustment, particularly with respect to cardiometabolic risk, socioeconomic status, and physiological data. These methodological variations may have contributed to heterogeneity in reported estimates of CV risk.

Recent evidence from randomized controlled trials and meta-analyses have significantly contributed to the understanding of CV risk associated with testosterone therapy. The TRAVERSE trial, a randomized, placebo-controlled study involving men aged 45 to 80 years with preexisting CVD or elevated CV risk, did not report a significant increase in MACE over a mean follow-up period of 33 months (HR 0.96; 95% CI, 0.78-1.17) [14]. Despite several strengths of the trial, including its large sample size, rigorous design, adjudication of CV outcomes, as well as detailed capture of CV risk factors at baseline, the TRAVERSE trial had a number of limitations. The trial experienced a high dropout rate, with more than 60% of participants both in testosterone and placebo groups discontinuing testosterone or placebo before completing the trial. Consequently, the duration of treatment exposure was limited to under 22 months, a much shorter exposure time than in the present study. Further, the outcome data was available for only about 80% of the possible follow-up time for both the testosterone and placebo groups. Additionally, the trial exclusively used transdermal testosterone formulations, which limits the generalizability of the findings to other commonly used forms of testosterone, such as intramuscular injections. This is contrasted by the present study, which includes various forms of testosterone (intramuscular injections, transdermal, and oral) with subgroup analysis for injectable testosterone and transdermal testosterone. Layton et al [32] report from their study that various forms of testosterone may have different CV risk profiles in the short term.

In the present study, both injectable testosterone and transdermal testosterone were associated with an increased risk of MACE; however, the association for injectable testosterone did not reach statistical significance. This difference may reflect pharmacokinetic differences between formulations or preferential prescribing of transdermal preparations in individuals perceived to be at higher CV risk. However, as this may be a consequence of limited statistical power, it should be interpreted with caution.

The TestES study, a meta-analysis of 35 randomized controlled trials, including 5601 participants with a mean age of 65 years [9] included individual participant data from 17 studies and 3431 participants, allowing for more precise estimates of CV risk. In this meta-analysis, testosterone therapy did not significantly differ from placebo in CV events (7.5% testosterone group vs 7.2% placebo; odds ratio 1.07; 95% CI, 0.81-1.42). Mortality rates were also similar (0.4% testosterone vs 0.8% placebo; odds ratio 0.46; 95% CI, 0.17-1.24). However, this meta-analysis also had a number of limitations, including the relatively short median follow-up duration of 9.5 months across the included trials. Furthermore, despite attempts to mitigate via subgroup and sensitivity analyses, heterogeneity in study designs, baseline CV risk factors, testosterone formulations, and treatment durations may have influenced the consistency and applicability of these results.

The present study has several strengths that build on the knowledge provided by the aforementioned research. The use of a large, regional-based cohort and longitudinal data from the West of Scotland Safe Haven allowed comprehensive tracking of patient exposures and CV outcomes. The relatively prolonged exposure to testosterone (≥2 years) and the extended follow-up (compared to several earlier studies) provide valuable insights into the CV risks, particularly in real-world clinical settings where patients may receive therapy lifelong. Additionally, the present study includes subgroup analyses for different formulations of testosterone. Furthermore, the detailed, linked electronic health records used in this study helped to minimize recall bias, a limitation that often affects observational studies relying on patient self-reporting.

Nevertheless, several limitations inherent in a retrospective analysis of this type must be acknowledged. First, our cohort only includes men aged 51 years and older. This was deemed suitable given that testosterone therapy is much more prevalent in middle-aged and older men. Mulligan et al [33] report that close to 40% of men aged 45 years and older are hypogonadal, and this population of hypogonadal men are likely to be receiving long-term testosterone therapy.

The reliance on prescription data alone to define testosterone exposure may have led to some misclassification, as electronic medical records are subject to several sources of measurement error [34]. We were also unable to assess prescribing rationale or adherence to therapy. Furthermore, testosterone is increasingly being acquired without prescriptions and used without appropriate medical oversight or indication [35], potentially contributing to exposure misclassification.

Another limitation is the lack of time-varying covariates, which restricted our ability to evaluate changes in testosterone dose or formulation over time. This may have masked dose-dependent effects on CV risk [36]. Additionally, key physiological data, such as testosterone concentrations and hematocrit, were not available, limiting mechanistic interpretation [37].

Although baseline data were available, we were unable to longitudinally evaluate several CV risk factors, including blood pressure, glycemic status, and lipid concentrations. Similarly, we were unable to account for the potential influence of cardioprotective medications as prescribing data were not available at sufficient resolution. We were also unable to assess the frequency of clinical encounters, which may have differed between groups and could have influenced the ascertainment of comorbidities or outcomes. This may have contributed to the observed differences in MACE. We also acknowledge the potential for residual confounding by unmeasured variables such as body mass index. Men with obesity often have lower total testosterone and sex hormone–binding globulin concentrations, which may lead to testosterone prescribing in the absence of true hypothalamic-pituitary-gonadal axis dysfunction. These individuals may also have an elevated baseline risk of CV events related to an adverse metabolic phenotype [38]. Although we adjusted for comorbidities commonly associated with cardiometabolic risk, including diabetes, hypertension, and liver disease, these adjustments may not fully account for the influence of adiposity on CV outcomes. Other important factors, including smoking history and unmeasured comorbidities not captured in this study, could not be adjusted for, and residual confounding remains a possibility. Such limitations could have biased associations toward the null in the case of exposure misclassification, or in either direction, where residual confounding from unmeasured factors such as body mass index and smoking was present.

Ethnicity was included as a covariate in our models; however, ethnicity data were incomplete for individuals without prior hospitalization. Including “unknown” as a category may have limited our ability to fully adjust for differences in CV risk by ethnicity, although sensitivity analyses yielded broadly similar results. A further limitation with our ethnicity data was that other ethnicities that were not “White” were grouped together as a “non-White” group. Moreover, although our cohort included a broad socioeconomic spectrum based on the distribution of SIMD quintiles, the single-region setting limits the generalizability to other populations, particularly those with different distributions of ethnicity.

Last, although the findings raise concern about long-term CV safety in older men receiving testosterone, the observational nature of this study precludes causal inference despite covariate adjustment. Also, the modest size of the testosterone-exposed cohort may have constrained statistical power in subgroup analyses and limited the broader applicability of the findings.

### Conclusion

While recent studies, including TRAVERSE and TestES, have provided important evidence regarding the short- to medium-term CV safety of testosterone therapy, considerable uncertainties remain about its long-term effect, particularly in real-world settings. The present study demonstrates an increased risk of MACE with testosterone therapy in older men, underscoring the need for extended follow-up beyond what has been previously reported. Future research should focus on longer-term observational studies and clinical trials that explore different testosterone formulations, including intramuscular testosterone, to determine whether formulation-specific risks exist in the long term. Furthermore, studies investigating time-varying effects, dosage alterations, and the role of biomarkers are essential to provide a comprehensive understanding of testosterone’s CV safety profile. Such data will be crucial in guiding evidence-based recommendations for testosterone therapy in clinical practice and ensuring the CV health of patients.

## Data Availability

Datasets used for specific West of Scotland Safe Haven projects may be made available on request, with appropriate ethical permissions and in accordance with standard Safe Haven security policies and procedures.

## Abbreviations

COPD: chronic obstructive pulmonary disease
CVD: cardiovascular disease
GGC: Greater Glasgow and Clyde
HR: hazard ratio
ICD-10: International Statistical Classification of Diseases and Related Health Problems, 10th Revision
MACE: major adverse cardiovascular events
NHS: National Health Service
SIMD: Scottish Index of Multiple Deprivation
TestES: Testosterone and Cardiovascular Events Study
TRAVERSE: Testosterone Replacement Therapy for Assessment of Long-term Vascular Events and Efficacy Response in Hypogonadal Men
